# Primary extrahepatic hydatid cyst of the thigh, an unusual presentation of sciatica: A case report of a diagnostic challenge

**DOI:** 10.1016/j.ijscr.2024.109545

**Published:** 2024-03-20

**Authors:** Ahmed Zendeoui, Mohamed Amine Gharbi, Mouadh Nefiss, Mohamed Hedi Ezzine, Anis Tborbi, Ramzi Bouzidi

**Affiliations:** Orthopaedic Department, Mongi Slim Hospital, Tunisia

**Keywords:** Hydatid cyst, Sciatica, Soft tissue location, Case report

## Abstract

**Introduction:**

Sciatica, a condition characterized by pain along the sciatic nerve distribution, is commonly associated with nerve compression or irritation. However, its etiology can vary, including rare non-spinal causes such as hydatid cysts. We present a case of hydatid cyst in the thigh causing sciatica-like symptoms, highlighting the diagnostic challenges and management approach.

**Case presentation:**

A 40-year-old patient with a history of pulmonary tuberculosis presented with persistent lumbosciatic pain despite conservative treatment. Physical examination revealed left sciatica without spinal abnormalities. MRI revealed a hydatid cyst in the thigh, causing nerve irritation. Surgical resection of the cyst was performed, achieving symptom resolution.

**Discussion:**

Hydatid cysts in skeletal muscles are rare, with atypical presentations complicating diagnosis. Localization in the thigh, particularly the biceps femoris muscle, is uncommon. Diagnostic modalities include imaging and serological tests, while treatment involves surgical excision and postoperative albendazole therapy.

**Conclusion:**

Recognition of rare presentations like thigh hydatid cysts causing sciatica-like symptoms is crucial for timely diagnosis and management. This case emphasizes the importance of considering unusual etiologies in refractory sciatica cases and underscores the complexity of medical diagnosis. Increased awareness among healthcare providers can lead to improved patient outcomes and prevent diagnostic delays.

## Highlights

• Hydatid cyst in thigh mimics sciatica
• Diagnostic challenge due to uncommon location delays recognition
• Surgical removal crucial for recovery
• Awareness needed for recognizing atypical symptoms

## 1 Introduction

Sciatica is a frequently incapacitating condition characterized by the presence of pain that follows the distribution of the sciatic nerve. It is often associated with compression or irritation of the nerve or its roots, although its etiology can vary widely and may include both spinal and non-spinal sources [1].

Our case involves the hydatid cyst, a rare parasitic infection caused by the larval stage of the Echinococcus tapeworm [2]. Although commonly found in the liver and lungs [3], the occurrence of a hydatid cyst in the limb soft tissues is an uncommon presentation, accounting for less than 2 % of all reported cases [4].

The lack of classical symptoms, coupled with the rare anatomical location, often leads to misdiagnosis or delayed treatment.

In this article, we present a case of a primary hydatid cyst of the posterior thigh, with an atypical clinical presentation which is a non-spinal cause of sciatica. We will illustrate the clinical features, diagnostic approach, and management modalities, of a such exceptional occurrence.

This case report has been reported in line with the SCARE Criteria [5].

## 2 Case presentation

We present the case of a 40-year-old male patient with a history of pulmonary tuberculosis in 2001, which was treated at a regional hospital with a four-drug antitubercular therapy for 6 months, resulting in complete remission. It's worth noting that the patient had a family history of tuberculosis and a history of consuming raw milk; Furthermore, there is no history of contact with animals, nor any previous contamination by hydatid cyst of the liver or any other location. This patient initially sought consultation due to a 10-month-long lumbosciatic pain that was unresponsive to medical treatment and well-conducted rehabilitation. The patient's examination revealed paroxysmal left sciatica in the L5-S1 region. The spine was flexible, with a Schober's index of +4 cm and a fingertip-to-floor distance of 1 cm. There was no tenderness upon palpation of the spinous processes, negative evidence of fracture, and a negative Lasègue and Leri signs. The hip, knee, and ankle joints showed full mobility, without local signs of inflammation.

**Fig. 1:**
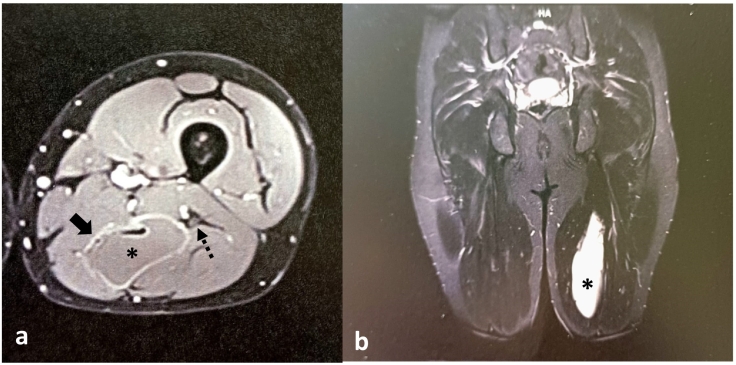
a: Axial cross-sectional image passing through the cyst using T1-weighted imaging with Gadolinium contrast, demonstrating the relationship of the cyst with neural structures. Solid arrow: cyst wall; dashed arrow: sciatic nerve; *: Hydatid Cyst. b: Frontal section in T2-weighted imaging with fat saturation, revealing the hydatid cyst involving the biceps femoris muscle.

The patient had consulted on multiple occasions, and the consistently given diagnosis was lumbosciatica, treated in the same manner with painkillers, anti-inflammatories, and rehabilitation. Our approach included further radiological exploration through an MRI of the lumbar spine. During the MRI, an incidental finding of a hydatid cyst on the posterior aspect of the thigh was made. Subsequently, an MRI of the thigh was performed (Fig. 1) confirming the diagnosis and providing a lesion map. The identified lesion was a well-defined cystic formation located on the medial aspect of the biceps femoris muscle, measuring 16 cm along its longest axis and 5 cm in the transverse plane. The cyst wall appeared thin and did not enhance after contrast injection.

**Fig. 2:**
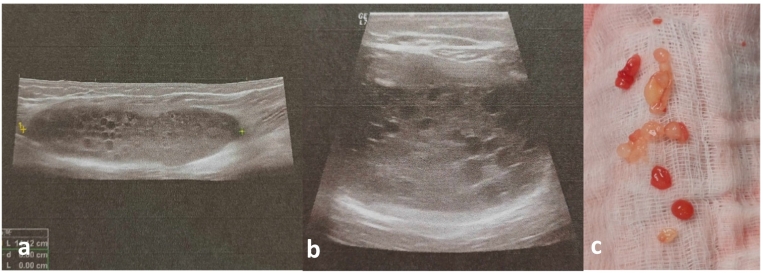
a.b: Ultrasound of the posterior aspect of the thigh showing an intramuscular formation with a well-defined wall containing rounded fluid-filled structures resembling daughter cysts of a hydatid cyst. c: Daughter hydatid cysts corresponding to the ultrasound image of rounded intracystic liquid formations.

An ultrasound of the posterior aspect of the thigh was subsequently performed, showing an intramuscular formation with a well-defined wall containing rounded fluid-filled structures resembling daughter cysts of a hydatid cyst (Fig. 2.a.b).

This localization in close proximity to the sciatic nerve led to nerve irritation, which was the cause of the symptoms resembling lumbosciatica. The irritation of the sciatic nerve explained the atypical clinical presentation. Consequently, we proceeded with a surgical resection of the entire cyst.

**Fig. 3:**
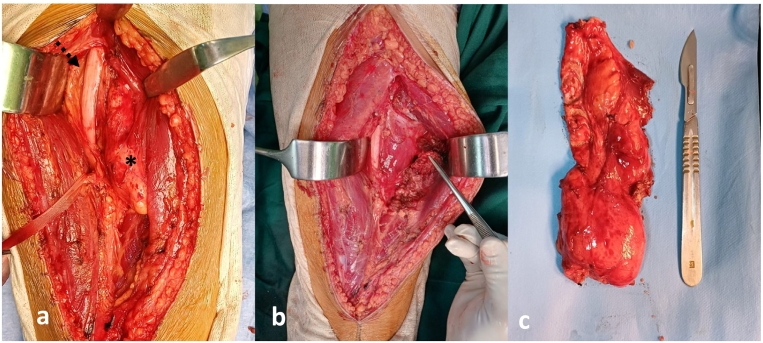
a: Identification of the hydatid cyst and its lateral association with the sciatic nerve, and medial connection with the semimembranosus muscle. *: Hydatid Cyst; dashed arrow: sciatic nerve. b: Specimen after en-bloc resection of the hydatid cyst along with the muscle attachments of the semimembranosus muscle, with the sciatic nerve being free. c: Hydatid cyst resected along with the tendon of the semimembranosus muscle.

Preoperatively, a variety of biological tests were conducted, ruling out any concurrent infection. It is noteworthy that the complete blood count (CBC) did not show eosinophilia, with an eosinophil concentration of 430 cells per mm^3^. We proceeded then with a surgical resection of the cyst. The patient underwent surgery in a prone position. A posterior approach to the thigh was performed, and passage through the intermuscular septum between the biceps femoris and semitendinosus muscles allowed exposure of the cyst and identification of the sciatic nerve (Fig. 3.a). The initial step involved neurolysis of the sciatic nerve and its release from the medial adhesions with the cyst, which was intimately related to the sciatic nerve over a length of at least 10 cm.

We found that the cyst was developed at the expense of the semimembranosus muscle, and its excision necessitated sacrificing the tendon of the semimembranosus muscle, along with a portion of muscle attached to the cyst to prevent its rupture and the spread of vesicles (Fig. 3.b). Exploration of the cystic cavity after resection revealed daughter cysts resembling the ultrasound appearance described preoperatively (Fig. 2.c). The resected specimen was sent for histopathological examination which confirmed the diagnosis (Fig. 3.c). Thorough irrigation with hypertonic saline solution was performed, and the patient was postoperatively prescribed Albendazole at a dose of 800 mg per day for a period of 1 month.

The patient's progress was marked by the timely healing of the surgical incision and the alleviation of sciatic pain. A three-month rehabilitation program, including muscle strengthening was prescribed. At the latest follow-up, eight months post-surgery, the patient reported complete resolution of pain and a successful return to work without difficulties.

## 3 Discussion

Hydatidosis, also known as echinococcosis, remains a major health concern in many parts of the world, especially regions with close contact between humans, livestock, and dogs, which serve as the definitive hosts for the parasite. The larvae are ingested through contaminated food or water, and they travel through the bloodstream, settling predominantly in the liver and lungs. However, in rare instances, the cysts can localize in various other organs, including the muscular system [1].

Primary skeletal muscle hydatidosis without involvement of the thoracic and abdominal organs is extremely rare. However, cases involving the muscles of the vastus lateralis [6], supraspinatus [7], biceps brachii [8], pectoralis major [9], gracilis [10] and quadriceps [11] have been reported in the literature. To our knowledge, there are no reported cases in the literature describing muscular localization in the biceps femoris muscle on the posterior aspect of the thigh. The muscle is considered as unfavorable site for hydatidosis because of its high lactic acid level [12].

The clinical presentation is not common; several forms may be observed. In the vast majority of cases, it is an assymptomatic cyst; in other cases, it presents as a painful, palpable mass slowly evolving and gradually increasing in size [10], limited mobility can be a revealing cause; Large cysts can restrict the movement of the muscle. Rupture or leakage of the cyst can trigger an immune response and lead to allergic reactions. In some cases -as in ours- the cyst's presence can cause compression of adjacent structures, leading to symptoms like nerve compression, which may cause numbness or weakness.

Compression of the sciatic nerve in our case, causing paresthesia of the dorsal aspect of the ankle radiating to the leg and foot, thus causing a symptomatology almost similar to lumbisciatica, was the reason for the diagnostic delay.

The radiological assessment includes ultrasound of soft tissues and radiographs as the first steps; ultrasound can diagnose the cyst size and characteristics; additional CT and MRI scans are generally necessary to study the rapport with neighbouring soft tissues.

Serological tests can help in the diagnostic investigation, Enzyme-linked immunosorbent assay (ELISA) and Western blot tests are commonly used to detect specific antibodies against the Echinococcus granulosus parasite.

The surgical removal of the cyst is the mainstay of treatment. The procedure aims to remove the entire cyst without rupturing it, as cyst rupture can lead to the dissemination of parasite material and potentially life-threatening complications.

To reduce and minimize the likelihood of recurrence, albendazole-based antiparasitic treatment is prescribed postoperatively: As recommended by the World Health Organization (WHO), albendazole is given in daily doses of 10–15 mg/kg body weight in two divided doses for at least 3–6 months [13].

The prognosis following cyst surgical resection depends on several factors, including its extension, the success of the surgical procedure, and the patient's overall health. If the cyst is completely removed without rupture and proper medical management is provided, the prognosis is generally favorable.

However, it's important to note that there can be potential complications associated with hydatid cyst surgery, such as infection, recurrence, or damage to surrounding tissues or nerves. Additionally, in some cases, there might be residual effects from nerve irritation that occurred prior to the surgery. Close post-operative follow-up and appropriate medical care are crucial to ensure the best possible outcome and to address any potential complications that may arise.

## 4 Conclusion

We shed light on the significance of recognizing and understanding the atypical presentation of a hydatid cyst located in the thigh. The particular manifestation of the cyst as lumbosciatalgia demonstrates how an unusual anatomical location, combined with nerve proximity, can result in an intricate clinical scenario. The delayed diagnosis highlights the challenges when presented with symptoms that deviate from the norm. Consequently, raising awareness among healthcare professionals about such rare presentations is crucial for timely and accurate diagnosis, ensuring appropriate management and avoiding delays in diagnosis and treatment. This case serves as a reminder of the complexity inherent in medical diagnosis and the necessity of maintaining a comprehensive differential diagnosis approach.

## Consent statement

Written informed consent was obtained from the patient for publication of this case report and accompanying images. A copy of the written consent is available for review by the Editor-in-Chief of this journal on request.

## Provenance and peer review

Not commissioned, externally peer-reviewed.

## Ethical approval

Ethical approval for this study was provided by the Ethical Committee of Mongi Slim University Hospital, Marsa, Tunisia.

## Funding

This research did not receive any specific grant from funding agencies in the public, commercial, or not-for-profit sectors.

## Author contribution

Ahmed Zendeoui: original draft writing.

Mohamed Amine Gharbi: Data analysis.

Mouath Nefiss: Data verification.

Mohamed Hedi Ezzine: Paper editing.

Anis Tborbi: Supervision.

Ramzi Bouzidi: Paper validation.

## Guarantor

Ahmed Zendeoui.

## Declaration of competing interest

The author(s) declared no potential conflicts of interest.
